# FZD7 expression marks mammary tumor–initiating cells

**DOI:** 10.1073/pnas.2522465122

**Published:** 2025-11-11

**Authors:** Christina C. N. Wu, Naycari De Luna, Erin Hairston, Erin D. Jeffs, Ashley Key, Stephen R. Adams, Sunil J. Advani, Terry Gaasterland, Dennis A. Carson, Karl Willert

**Affiliations:** ^a^Department of Cellular & Molecular Medicine, University of California, San Diego, La Jolla, CA 92093-0695; ^b^Department of Medicine, University of California, San Diego, La Jolla, CA 92093; ^c^Department of Pharmacology, University of California, San Diego, La Jolla, CA 92093; ^d^Department of Radiation Medicine and Applied Science, University of California, San Diego, La Jolla, CA 92093; ^e^Marine Biology Research Division, Scripps Institution of Oceanography, University of California, San Diego, La Jolla, CA 92093-0202

**Keywords:** WNT, FZD, breast cancer, tumor organoids, antibody drug conjugate

## Abstract

The WNT signaling pathway has long been implicated in tumorigenesis across multiple cancer types, including breast cancer. However, the complexity arising from the large number of WNTs and their receptors has made it challenging to pinpoint specific components driving tumor development. Using the MMTV-*Wnt1* genetically engineered mouse model, which develops mixed-lineage mammary tumors resembling triple-negative breast cancer and composed of both basal and luminal subtypes, we identify the frizzled class receptor 7 (Fzd7) as a key player. Fzd7 is expressed on mammary tumor cells that show enhanced tumorigenic potential in both orthotopic transplantation and tumor organoid assays. Despite the cellular heterogeneity of MMTV-*Wnt1* tumors, treatment with a Fzd7-specific antibody–drug conjugate significantly suppresses tumor growth, suggesting that Fzd7-expressing cells are critical drivers of tumor progression. These findings show that Fzd7 marks a population of putative tumor-initiating cells and that targeting Fzd7 offers a promising therapeutic strategy for breast cancer.

## Significance

Triple negative breast cancer (TNBC) is an aggressive subtype with limited therapeutic options. We identify FZD7 as a key marker of tumor-initiating basal cells in the MMTV-*Wnt1* mouse model of TNBC and demonstrate its value as a therapeutic target. Using humanized Fzd7 mice, we show that a highly specific FZD7 antibody enables isolation of FZD7^+^ tumor cells with enhanced growth potential and selective sensitivity to a FZD7-antibody drug conjugate (ADC). Tumor-derived 3D organoids recapitulate in vivo complexity and provide a platform to evaluate FZD7-dependent growth and drug response. Translation of these findings to human TNBC cells underscores the clinical promise of FZD7-targeted ADC therapy, offering a potential approach for aggressive breast cancers lacking effective treatments.

The discovery of the first mammalian *Wnt* gene (then called *int1*) (1) has inextricably linked Wnt signaling to cancer. Since this seminal discovery, mutations in genes that encode components of Wnt signaling, including adenomatous polyposis coli (*APC*), β-catenin (*CTNNB1*), and *Axin*, as well as several Wnt target genes, have been identified in a variety of tumor types. Increased WNT signaling activity resulting from elevated cell surface levels of WNT receptors, such as through loss-of-function mutations of Wnt receptor-specific ubiquitin ligases ZNRF3 and RNF43 (2) and recurrent R-spondin (RSPO) fusion proteins (3), has also been documented in a number of cancers. Among WNT receptors, the gene frizzled class receptor 7 (*FZD7*) has garnered significant attention as being upregulated in a broad spectrum of solid tumors (4, 5), including breast cancer (6–8), ovarian cancer (9–11), hepatocellular carcinoma (12–15), Wilms’ tumor (16), gastric cancers (17, 18), pancreatic cancer (19, 20), and colon cancer (21, 22). FZD7 expression is also elevated in a variety of stem cell populations, including human pluripotent stem cells (23, 24) and intestinal stem cells (25–27), raising the possibility that cancer cells hijack functions of this receptor to establish tumor initiating potential.

Breast cancer is the most commonly diagnosed cancer and the leading cause of cancer-related death in women, with 2.3 million cases and 685,000 deaths reported globally in 2020 (28). Standard treatments include surgery, hormone therapy, chemotherapy, immunotherapy, and bone-modifying agents, but their effectiveness is often limited by adverse effects and resistance, highlighting the need for new therapies. Previously, we (10) and others (4, 6, 8) found *FZD7* expression to be elevated in triple-negative breast cancer (TNBC) compared to other subtypes, making it a promising therapeutic target.

Despite a well-established role for WNT signaling in cancer, including breast cancer, with impacts on proliferation, metastasis, stemness, treatment resistance, and the immune microenvironment, no treatments targeting this pathway have emerged. Here, we leveraged a genetically engineered mouse model, MMTV-*Wnt1*, to study the role of Fzd7 in mammary tumorigenesis. This mouse model, where a Wnt1 transgene is expressed under control of a MMTV transcriptional element, established *Wnt1* as an oncogene, capable of initiating and promoting mammary tumorigenesis (29). Tumors develop with variable latency from 2 mo to 1 y of age (29–31) and display extensive tumor cell heterogeneity with comingling basal and luminal subsets that resembles the complexity of human breast cancers (32) and exhibit features of TNBCs, including a similar transcriptional profile (33). These tumors express high levels of Fzd7 in the basal compartment (6), a cell population associated with breast cancer initiation. Furthermore, as previously demonstrated (6), these Fzd7 expressing cells mark not only tumor-initiating cells (TICs) but also mammary gland stem cells (MaSCs).

Here, we demonstrate that Fzd7-expressing tumor cells drive the aggressive growth of transplanted MMTV-*Wnt1* tumors and that selective targeting with a FZD7-specific antibody drug conjugate (ADC) blocks tumor growth.

## Results

Previously, we developed an antibody specific to human FZD7 (F7-Ab) that fails to react with mouse Fzd7, an observation critical in pinpointing the epitope of F7-Ab to a region between the extracellular cysteine-rich domain (CRD, where WNTs bind) and the first transmembrane domain (10, 34). Using CRISPR-Cas9-mediated gene editing, we generated an allele of mouse Fzd7 that produces a protein that is fully reactive with F7-Ab. We refer to mice carrying this allele as HF7 and the resulting protein as hFzd7. HF7 mice are phenotypically normal and exhibit no features associated with *Fzd7* loss of function (35), indicating that the hFzd7 protein is functional. To confirm Fzd7 expression in the MMTV-*Wnt1* tumors as previously demonstrated (6), we crossed the HF7 allele into MMTV-*Wnt1* mouse model (*SI Appendix*, Fig. S1 *A* and *B*). MMTV-*Wnt1* mice carrying the wild type (WT) or the HF7 allele of *Fzd7* developed mammary tumors with similar latencies (*SI Appendix*, Fig. S1*C*). Immunohistochemistry (IHC) with F7-Ab demonstrated that hFzd7 is detected only in tumors derived from HF7 mice, not from WT mice (*SI Appendix*, Fig. S2*A*). Flow cytometry (FC) detected hFzd7 protein in multiple independent HF7-derived tumors (*SI Appendix*, Fig. S1 *A–D*). Immunofluorescence (IF) staining (*SI Appendix*, Fig. S2*B*) and FC (*SI Appendix*, Fig. S2*C*) further demonstrated that hFzd7 is expressed in the basal compartment, as defined by Cd49f (gene name Itga6) coexpression, and not in the luminal compartment, marked by Epcam expression. In all three applications (IHC, IF, and FC), hFzd7 is only detected in tumors derived from HF7, not WT mice, confirming the specificity of the F7-Ab (*SI Appendix*, Figs. S1*D* and S2).

**Fig. 1.**
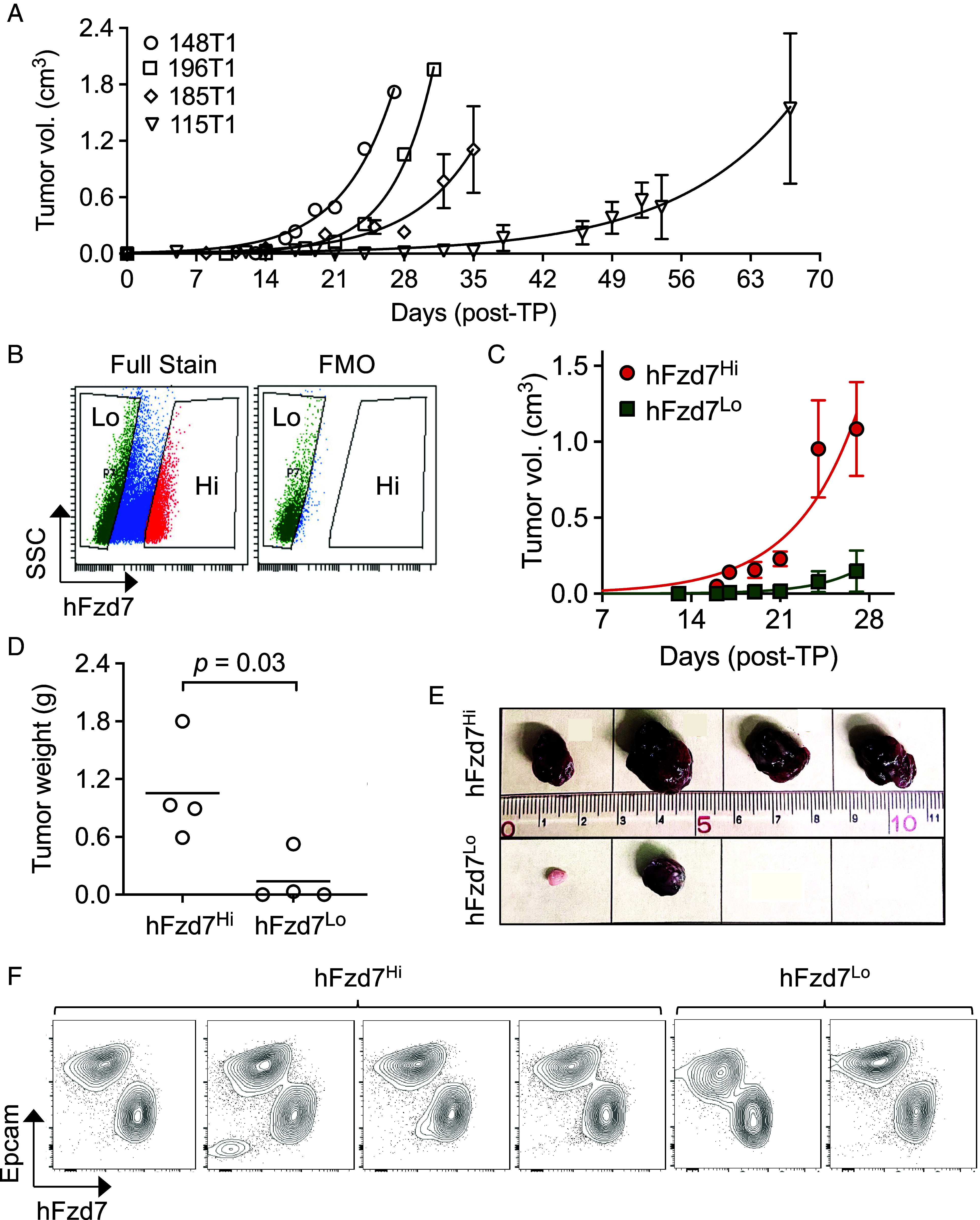
Fzd7-expressing cells harbor tumor-initiating potential. Tumor cells prepared from MMTV-*Wnt1*;HF7 mice were orthotopically transplanted into the 4th mammary fat pad of C57BL/6 either as bulk primary cells (*A*; 10^5^ cells/mouse, n = 1 to 4 mice) or as sorted hFzd7^Hi^ and hFzd7^Lo^ populations (*B*–*F*; 10^4^ cells/mouse, n = 4 mice). (*A*) Analysis of tumor growth kinetics using caliper measurements. Tumors were evaluated two to three times weekly until reaching maximum size. Symbols represent individual primary tumors. (*B*) Tumor cells (2 × 10^6^) were labeled with the fluorescently labeled F7-Ab and FACS sorted into hFzd7^Hi^ and hFzd7^Lo^ populations into the corresponding gates. FMO, fluorescence minus one (removal of F7-Ab) as background level. Bars indicate mean SEM. (*C*) Tumor growth kinetics in mice transplanted with hFzd7 sorted cells. (*D*) Assessment of tumor weights. *P < 0.05, unpaired two-tailed *t* test with Welch’s correction. (*E*) Images of tumors isolated from transplanted mice. Ruler shown in cm. (*F*) Flow cytometric analysis of tumors shown in panel *E* demonstrates the presence of luminal (Epcam positive) and basal (hFzd7 positive) cells. Data shown are representative of two independent studies with similar results.

Consistent with prior work (31, 36), MMTV-*Wnt1* tumors can be propagated through serial transplantation into recipient mice, with individual tumor lines exhibiting variable tumor growth kinetics (Fig. 1*A*). Having established that Fzd7 is expressed on a subset of tumor cells, the Cd49f-positive basal-like population, we next sought to investigate the tumorigenic potential of cells expressing high vs. low levels of hFzd7 (hFzd7^Hi^ and hFzd7^Lo^, respectively). Dissociated tumor cells were depleted for Cd45, which marks hematopoietic cells, sorted with fluorescently labeled F7-Ab to isolate hFzd7^Hi^ and hFzd7^Lo^ cell populations (Fig. 1*B* and *SI Appendix*, Fig. S3*A*) and then transplanted orthotopically into the 4th mammary fat pad of syngeneic mice. Using multiple primary tumors, we found that transplanted hFzd7^Hi^ cells grew faster and produced larger tumors than hFzd7^Lo^ cells (Fig. 1 *C*–*E* and *SI Appendix*, Fig. S3 *B*–*D*). Flow cytometric analyses of the tumors derived from hFzd7^Hi^ cells demonstrated that these tumors established a similar cellular composition as the original tumor, with comingling of hFzd7^Hi^;Epcam negative and hFzd7^Lo^, Epcam positive cells (Fig. 1*F*). Furthermore, the smaller tumors arising in mice injected with Fzd7^Lo^ cells (two of four mice) likewise established this similar tumor heterogeneity (Fig. 1 *E* and *F*), suggesting that either the flow cytometric separation of hFzd7^Hi^ and hFzd7^Lo^ cell populations is imperfect, or Fzd7^Lo^ cells can give rise to Fzd7^Hi^ cells. Secondary serial transplantation produced tumors with similar kinetics, and hFzd7^Hi^ tumors developed faster and grew bigger than hFzd7^Lo^ tumors (*SI Appendix*, Fig. S3 *E*–*G*). These experiments demonstrate that the basal cell population expressing Fzd7 harbor higher tumorigenic potential than cells expressing low levels of Fzd7. In addition, hFzd7^Hi^ cells produced tumors with a similar cellular heterogeneity as the original tumor.

**Fig. 2.**
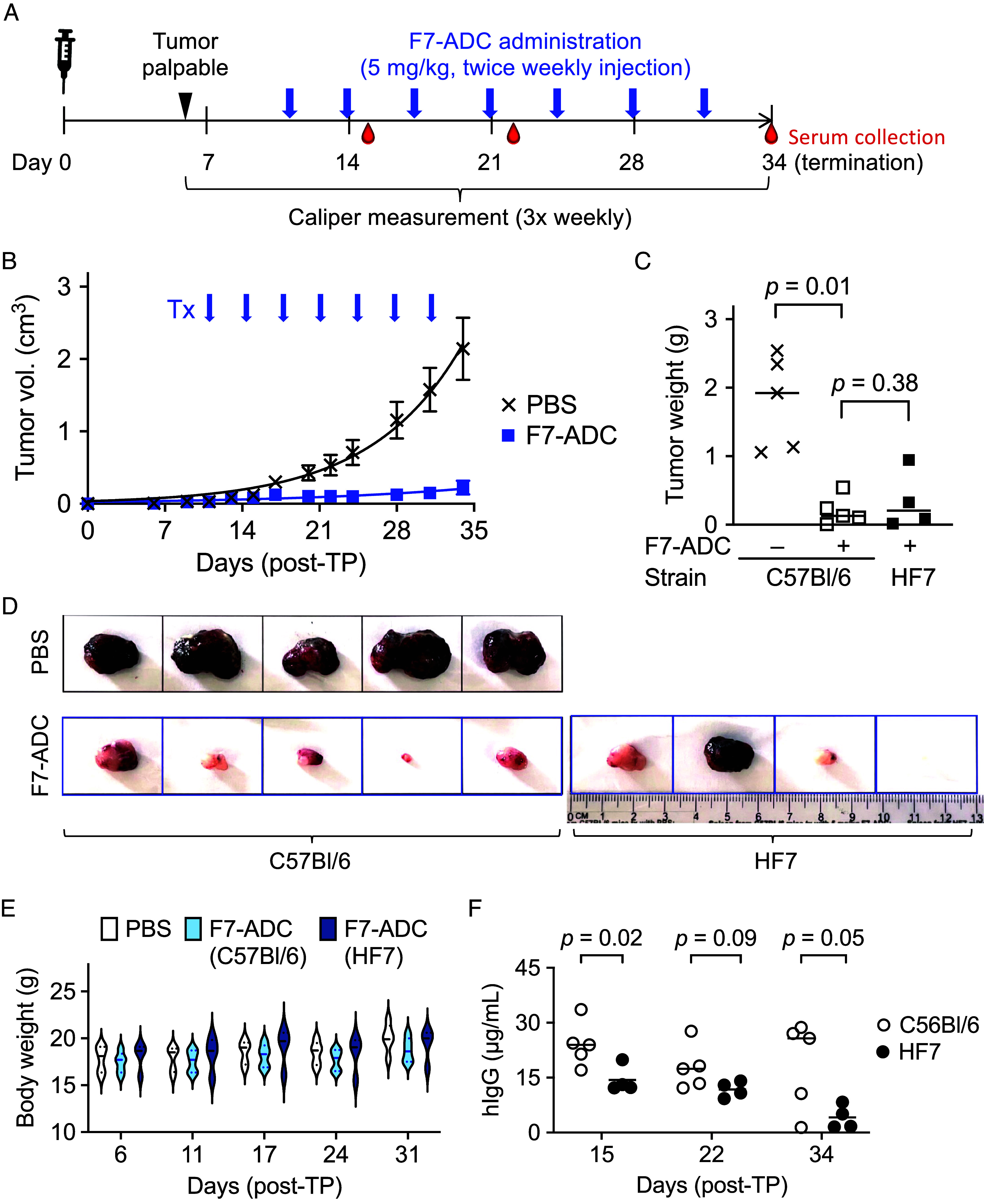
A FZD7-specific ADC inhibits tumor growth. (*A*) Schematic of the F7-ADC dosing strategy in serially transplanted mouse tumor model. Tumor cells (10^5^ cells/mouse) were orthotopically transplanted into C57BL/6 and HF7 mice. Tumor-bearing C57BL/6 mice were stratified into two groups based on measurable tumor volume and body weight for treatment with either PBS or 5 mg/kg F7-ADC (seven doses in total). (*B*) Tumor volumes were evaluated by caliper measurements of mice treated with either PBS (C57BL/6 n = 5) or F7-ADC (C57BL/6 n = 5 and HF7 n = 4). Error bars indicate SEM. (*C*) Assessment of tumor weights. *P < 0.05, paired two-tailed student’s *t* test. (*D*) Images of tumors isolated from treated mice. (*E*) Assessment of body weight at different time points. There were no observable behavioral changes in animals throughout treatments and no statistical significant difference in body weights by Brown–Forsythe ANOVA test. (*F*) Systemic F7-ADC levels in sera collected at 24 h after the 2nd and 4th doses and at termination were assessed by human IgG1 ELISA. Little or no hIgG1 were detected from PBS-treated sera. *P < 0.05, paired two-tailed student’s *t* test.

Having established that Fzd7-positive cells harbor tumor initiating potential, we reasoned that targeted killing of these cells with a Fzd7-specifc antibody drug conjugate (F7-ADC) composed of F7-Ab conjugated to an antimitotic payload drug, monomethyl auristatin E (MMAE), would lead to tumor growth arrest. Previous experiments established that this ADC blocked growth of ovarian cancer cells in vitro and in vivo (10). Mice carrying transplanted MMTV-*Wnt1* tumors were injected with this F7-ADC twice per week over 4 wk (Fig. 2*A*). Tumor growth was reduced in mice treated with the F7-ADC compared to mice injected with vehicle (Fig. 2 *B*–*D*). To demonstrate that F7-ADC exhibited no on-target toxicity, we included in this study a cohort of four mice homozygous for the HF7 allele of Fzd7. As in WT mice, the ADC blocked tumor growth in HF7 mice while producing no overt adverse side effects, such as loss of body weight (Fig. 2*E*). The presence of F7-ADC was detected in mouse serum samples using a human IgG-specific ELISA throughout the 4-wk treatment period, with a slight reduction in hIgG concentrations detected in serum from HF7 mice compared to WT mice (Fig. 2*F*), suggesting that the hFzd7 protein acts as a sink for the F7-ADC. These data indicate a favorable safety profile for F7-ADC, consistent with our previous study in which HF7 mice were treated acutely with a high dose of ADC (10).

To expand on these mouse studies, we sought to develop an in vitro cell culture system that recapitulates the cellular complexity of the in vivo tumor cells. Using established protocols (37, 38) with some modifications, we established three-dimensional (3D) tumor organoids from dissociated MMTV-*Wnt1*;HF7 tumor cells. Over the course of 7 d, these tumor cells grew into spherical structures of up to 100 to 200 μm in size (*SI Appendix*, Fig. S4 *A* and *B*). Immunofluorescent staining of these organoids revealed these structures to be cystic and encapsulated by hFzd7-expressing cells (*SI Appendix*, Fig. S4*C*). Dissociation of tumor organoids followed by FC revealed a similar cellular composition as the whole tumor, with a population expressing Epcam (the luminal compartment) and a population expressing Cd49f and hFzd7 (the basal compartment, see *SI Appendix*, Fig. S4*D*). As expected, these tumor organoids lacked cells expressing the hematopoietic marker Cd45, indicating the absence of tumor vasculature, which requires an in vivo environment to develop.

In parallel, we also attempted to grow these tumor cells in a two-dimensional (2D) setting. Although we were able to derive tumor cell lines that could be propagated over multiple passages in a 2D environment, hFzd7 expression was highly variable, with some lines expressing high hFzd7 and others expressing no detectable hFzd7 (*SI Appendix*, Fig. S4 *D* and *E*). In addition, these cell lines lacked the cellular heterogeneity observed in the tumor organoid system, with cells expressing either the luminal marker Epcam or the basal marker hFzd7 (*SI Appendix*, Fig. S4*D*), but never both. Together, these data demonstrate that in contrast to 2D settings, a 3D organoid culture milieu recapitulates aspects of the whole tumor and may serve as a robust platform to further interrogate tumor growth in vitro.

**Fig. 3.**
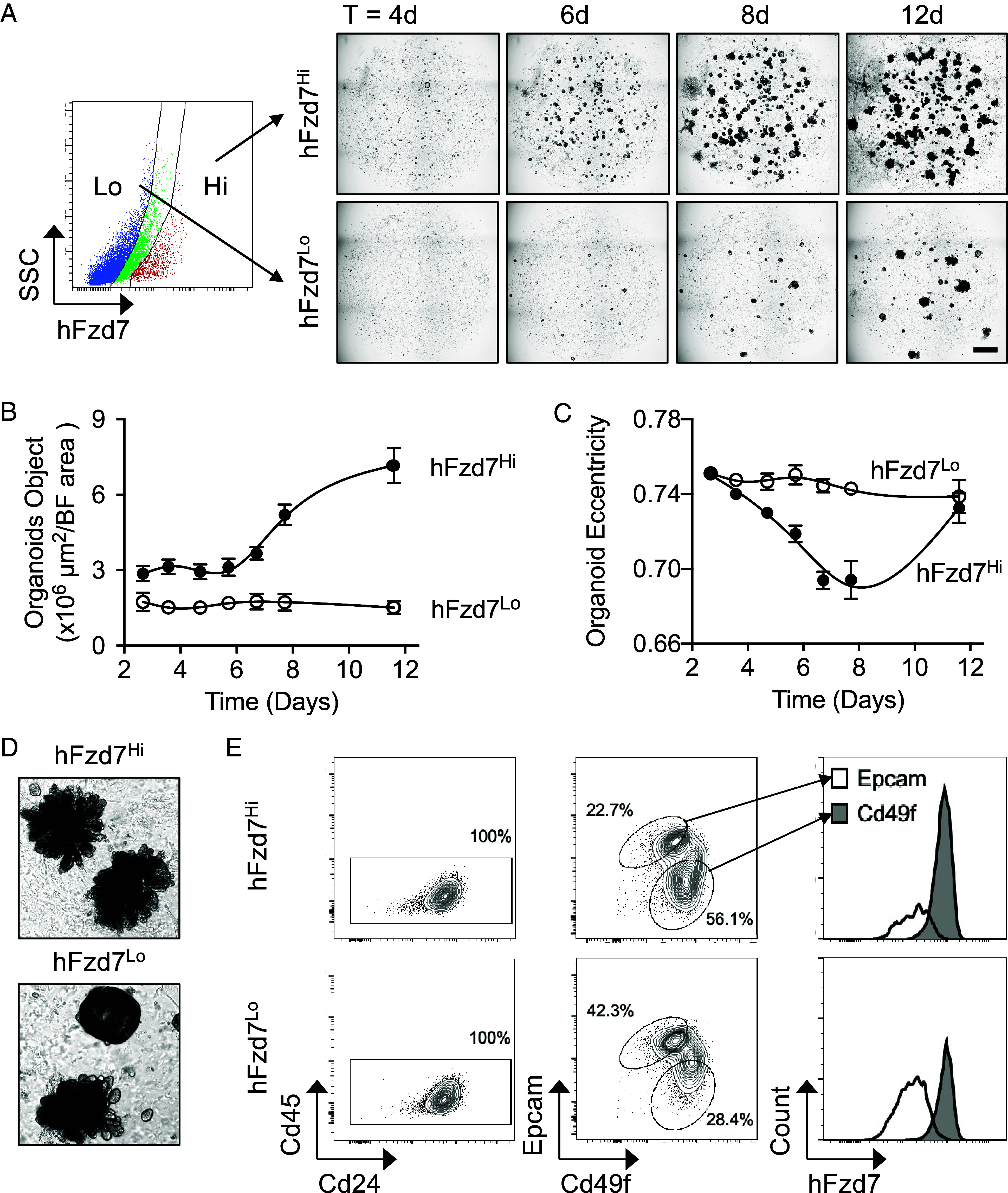
Fzd7-expressing cells promote organoid growth. (*A*–*C*) MMTV-*Wnt1*;HF7 tumor cells depleted for Cd45 and sorted into hFzd7^Hi^ and hFzd7^Lo^ populations were cultured in 3D organoid conditions (1,000 cells/Matrigel dome, n = 3). Organoid growth was monitored over 7 d using an incubated microscope (IncuCyte®). (*A*) Time lapse of a representative dome with organoid growth at 4× magnification. (Scale bar, 0.8 mm.) Assessment of organoid growth by quantification of total object area per total bright field area (*B*) and of organoid shapes by eccentricity (*C*). Error bars indicate SEM. Reduction in eccentricity was observed in domes seeded with hFzd7^Hi^ cells indicating organoids were forming and becoming more rounded. (*D*) Images of representative organoids in long-term culture (31 d). (*E*) FC analysis of dissociated tumor and organoid cells demonstrating that organoid cellular composition resembles that of the original tumor. Data shown are representative of two independent experiments.

Next, we used sorted hFzd7^Hi^ and hFzd7^Lo^ cells from dissociated tumors to establish organoid cultures. Both cell populations produced tumor organoids; however, the hFzd7^Hi^ population more rapidly expanded into organoids than the hFzd7^Lo^ population (Fig. 3 *A* and *B*), a finding consistent with our observation that hFzd7^Hi^ cells grew faster and into larger tumors than hFzd7^Lo^ cells upon transplantation (Fig. 1*C* and *SI Appendix*, Fig. S3*C*). In addition, growth of hFzd7^Hi^ organoids underwent a transient decrease in eccentricity, a metric of organoid roundness (Fig. 3*C*), indicating that these cells produce more structurally complex organoids than their hFzd7^Lo^ counterparts, a difference reflected in their morphology (Fig. 3*D*). Flow cytometric analysis revealed that these hFzd7^Hi^ and hFzd7^Lo^-derived organoids exhibit the same tumor heterogeneity as tumor cells, with luminal (Epcam-positive) and basal compartments (Cd49f and hFzd7-positive) (Fig. 3*E*). As expected, hFzd7^Hi^-derived organoids contained a larger fraction of basal cells compared to hFzd7^Lo^-derived organoids (56.1% vs. 28.4%, respectively). Together with the in vivo transplantation studies, these experiments support our hypothesis that Fzd7-expressing cells harbor tumor initiating potential.

**Fig. 4.**
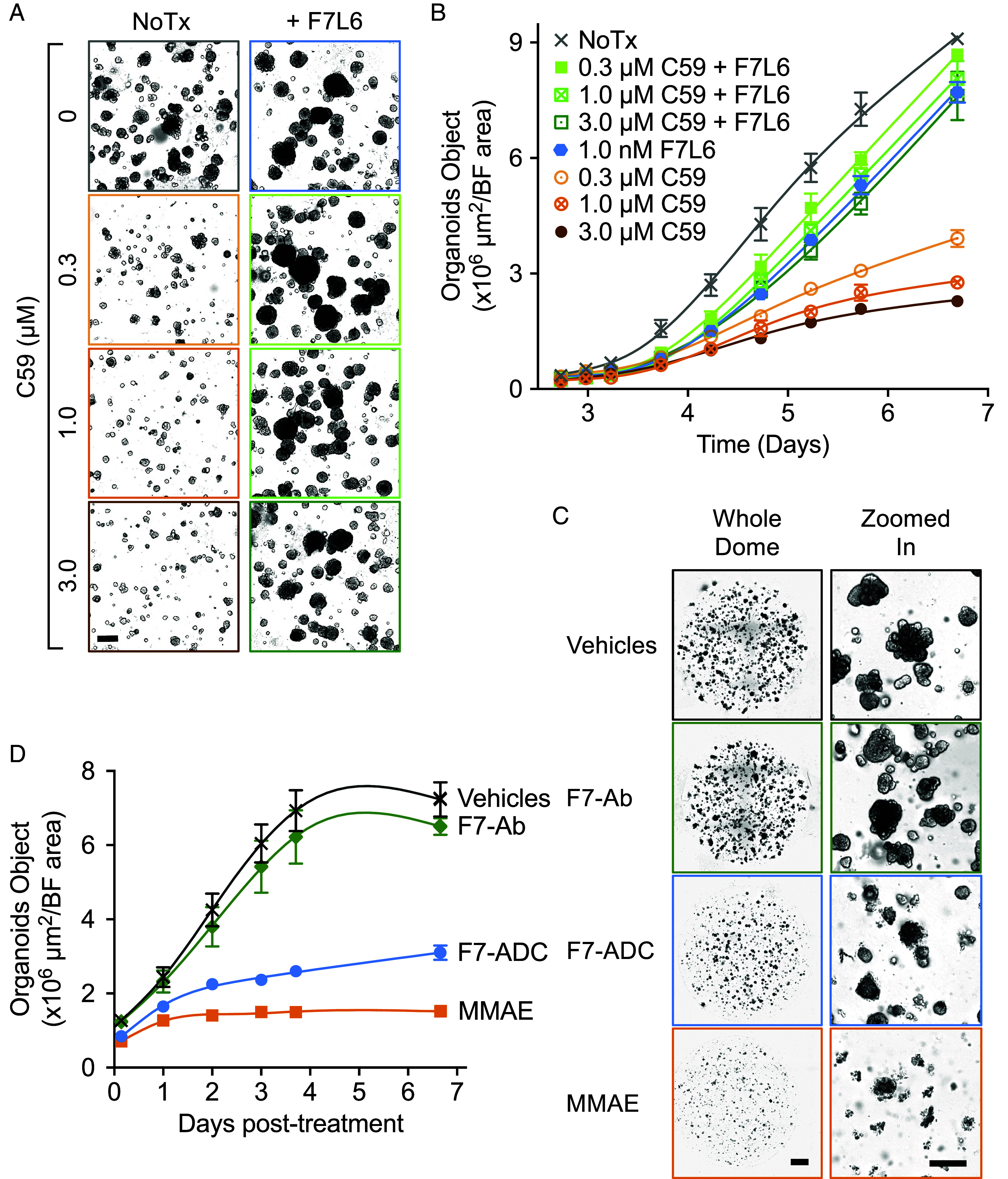
Organoid growth requires Fzd7 expression and signaling. MMTV-*Wnt1*;HF7 tumor cells were cultured in 3D organoid conditions (3,000 cells/Matrigel dome, n = 3), treated with either a PORCN inhibitor, C59, at day 0 (*A* and *B*) or F7-ADC at day 3 (*C* and *D*), and monitored using IncuCyte® for 7 d. (*A*) Representative images of organoids at the end of treatments, 4× magnification. F7L6, hFzd7/Lrp6-selective Wnt mimetic. NoTx, no treatment. (Scale bars, 0.3 mm.) (*B*) Quantitation of the kinetics of organoid growth shown in panel *A*. (*C*) Treatment of organoids with F7-ADC inhibits organoid growth. MMAE, the antimitotic drug conjugated to F7-Ab to generate F7-ADC, was used as a positive control for organoid growth inhibition and killing. [Scale bars, 0.8 mm (whole dome) and 0.2 mm (zoomed in).] (*D*) Quantitation of the kinetics of organoid growth shown in panel *C*. Error bars indicate SEM. Data shown are representative of two independent studies with similar results.

Tumorigenic growth in the MMTV-*Wnt1* model is driven by high levels of *Wnt1* expression, and treatment with inhibitors of Porcupine (Porcn), a protein essential for Wnt acylation and subsequent secretion, blocks tumor growth (39). Likewise, treating tumor organoids with the Porcn inhibitor C59 arrested their growth (Fig. 4 *A* and *B*), indicating that Wnt signaling is required for organoid growth. To test whether hFzd7 mediates such a growth signal, we employed a FZD7-selective Wnt mimetic, called F7L6, that potently activates Wnt/β-catenin signaling. In contrast to recombinant Wnt proteins, such as Wnt3a, which are notoriously promiscuous in their interactions with their receptors (40), F7L6 only engages human FZD7 (34) and mouse hFzd7 (10). F7L6 rescued the growth of C59-treated organoids (Fig. 4 *A* and *B*), demonstrating that signaling through hFzd7 was sufficient to promote organoid growth. F7L6 did not rescue the growth of tumor organoids derived from WT (non-HF7) MMTV-*Wnt1* tumors, confirming selectivity of F7L6 for human FZD7 and hFzd7, but not WT Fzd7 (*SI Appendix*, Fig. S5). As expected, Wnt3a rescued the growth of C59-treated organoids derived from WT and HF7 MMTV-*Wnt1* tumors (*SI Appendix*, Fig. S5).

To establish that hFzd7-expressing cells are critical for organoid growth, we treated organoid cultures with the aforementioned F7-ADC. As expected, organoids failed to grow when treated with F7-ADC (Fig. 4 *C* and *D*). Of note, treatment with F7Ab had no effect on organoid growth, demonstrating that the naked antibody does not interfere with hFzd7 to transduce endogenous Wnt signaling. Furthermore, organoids treated with either F7-ADC or MMAE show signs of cells bursting (Fig. 4*C*), suggestive of cell death. In contrast, C59-treated organoids remain small and fail to grow (Fig. 4*A*), suggesting that organoid growth is blocked, possibly through senescence or differentiation. These data demonstrate that the mammary tumor organoid culture system provides an alternative to labor-intensive and costly animal studies as described in Fig. 2.

**Fig. 5.**
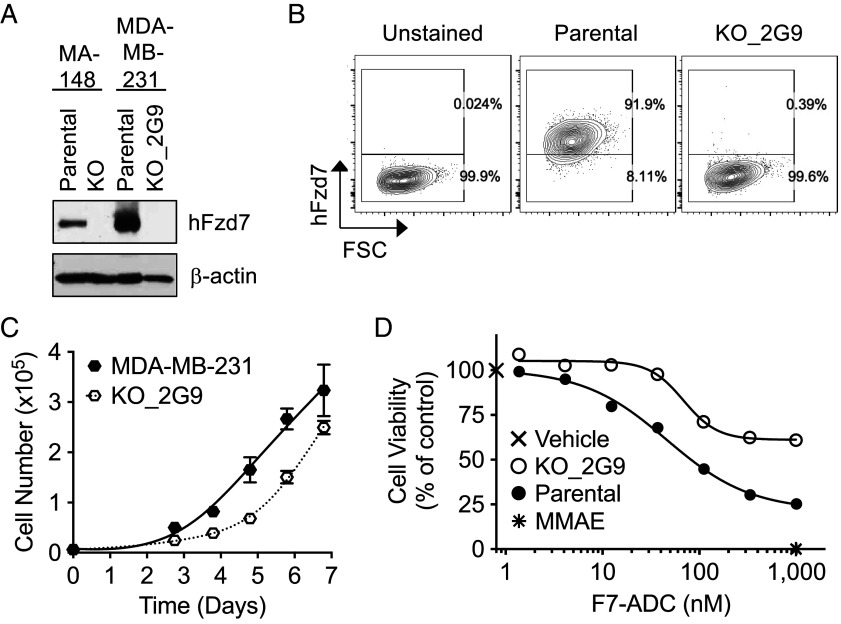
Growth inhibition of human TNBC cell line (MDA-MB-231) by F7-ADC. (*A*) Immunoblot of lysates from an ovarian cancer cell line, MA-148, a TNBC line, MDA-MB-231, and their corresponding FZD7 (KO) knockout lines. (*B*) FC demonstrates lack of FZD7 protein in the KO line 2G9. (*C*) Growth kinetic analysis indicates that the KO line grows slower than the parental MDA-MB-231 line. (*D*) Dose titration of F7-ADC on killing MDA-MB-231 clones. Vehicles, negative control; MMAE, positive control. Each symbol represents mean value from triplicate wells. Similar results are observed from three independent experiments.

To advance the above observations toward human studies, we examined FZD7 expression in human breast cancer cell lines. According to The Human Protein Atlas, the TNBC line MDA-MB-231 expressed high *FZD7* expression, which we confirmed by immuno-blotting (Fig. 5*A*) and FC (Fig. 5*B*). Levels of FZD7 were higher in MDA-MB-231 than in MA-148 (Fig. 5*A*), an ovarian cancer cell line that we used previously to demonstrate ADC killing in vitro and in vivo. To study the role of FZD7 in this TNBC cell line, we used CRISPR/Cas9-mediated gene editing to mutate the *FZD7* gene. Only one clone, 2G9, with no detectable FZD7 protein (Fig. 5 *A* and *B*) was isolated, which exhibited a slightly slower growth rate than the parental line (Fig. 5*C*). Treatment of MDA-MB-231 with the FZD7-specific ADC resulted in a significant reduction in growth of these cells, an effect that was reduced in the FZD7 knockout clone 2G9 (Fig. 5*D*). These data provide evidence that FZD7 is a good target to treat TNBC with F7-ADC.

## Discussion

In this study, we establish FZD7 as a defining marker of tumor-initiating basal cells in MMTV-*Wnt1*-driven breast cancer, a well-characterized model of TNBC, a highly aggressive breast cancer subtype with poor prognosis and high risk of recurrence and metastasis. Using genetically engineered mice carrying a humanized allele of Fzd7, we demonstrate that a FZD7-specific antibody (F7Ab) can reliably identify and isolate Fzd7-expressing tumor cells. These cells exhibit markedly enhanced growth potential in both transplantation assays and organoid culture, underscoring their functional role as TICs. Importantly, this population is selectively sensitive to a FZD7-directed antibody–drug conjugate (F7-ADC), establishing a therapeutic opportunity to target the basal cell compartment that drives tumor propagation. Complementary studies in 3D tumor organoids confirm these findings: Organoids derived from MMTV-*Wnt1* tumors preserve the cellular heterogeneity and lineage architecture of the in vivo tumors, with luminal and basal compartments clearly demarcated and Fzd7 expression restricted to basal cells. These organoids represent a robust in vitro platform for probing FZD7-dependent biology and faithfully recapitulate the therapeutic response to the F7-ADC observed in vivo. The extension of these findings to human TNBC cell lines further reinforces the translational potential of FZD7-targeted therapy in a disease context that lacks effective treatment options.

The high degree of specificity of F7Ab for FZD7 circumvents the challenges that limited prior FZD-directed approaches. Vantictumab (OMP-18R5), a pan-FZD antibody that reached clinical trials, demonstrated modest antitumor activity but was hindered by dose-limiting toxicities, particularly in bone, likely linked to its broad reactivity with multiple FZD family members (FZD1, 2, 5, 7, and 8) (41, 42). In contrast, F7Ab is highly selective for FZD7, suggesting a more favorable therapeutic window. Furthermore, the ADC format transforms F7Ab from a passive binder into a potent therapeutic agent, capable of delivering cytotoxic payloads specifically to FZD7-expressing tumor cells. Notably, administration of the F7-ADC in immune-competent mice produced robust antitumor effects without detectable toxicity, despite the known expression of FZD7 in certain adult stem cell populations, such as the intestine (25, 27). This finding supports the hypothesis that the efficacy of the F7-ADC derives from both tumor-selective overexpression of FZD7 and the strict specificity of F7Ab.

The organoid platform further enhances the translational relevance of these studies and offers an alternative to mouse models which have well-documented limitations (43, 44). Unlike conventional 2D cultures, MMTV-*Wnt1* tumor-derived organoids preserve the complexity of the original tumors, including basal-luminal organization and basal-restricted Fzd7 expression. Their parallel sensitivity to the FZD7-ADC compared with transplanted tumors underscores their value as a preclinical model for both mechanistic studies and drug evaluation. The lack of therapeutic effect in 2D tumor-derived cell lines, likely reflecting loss of cellular heterogeneity and acquisition of neoantigens, further highlights organoids as a superior in vitro system. Beyond this study, the ability of organoids to capture tumor architecture, cellular interactions, and lineage hierarchies positions them as an important platform for dissecting WNT/FZD signaling dynamics and for developing precision therapeutics.

Placing these results in the broader landscape of WNT/FZD-targeted therapies, several distinctions stand out. Unlike vantictumab, which binds the extracellular CRD of multiple FZDs and directly blocks WNT ligand binding (42), F7Ab recognizes a linker region between the CRD and the first transmembrane domain of FZD7 (34). This binding site does not interfere with WNT ligand engagement, and indeed, F7Ab has shown little evidence of blocking WNT signaling in prior studies. For example, the Fab fragment of F7Ab failed to inhibit Wnt3a signaling in human embryonic stem cells (23), and intact F7Ab had no effect on viability of several ovarian cancer cell lines (10). Similarly, in our organoid experiments, F7Ab alone did not alter tumor growth. These observations strongly suggest that the therapeutic value of F7Ab lies in its utility as a highly specific delivery vehicle rather than as a signaling antagonist. However, it remains possible that F7Ab has subtle, context-dependent effects on FZD7 activity, for example, by promoting receptor internalization or altering receptor turnover, and this possibility warrants further investigation.

The mechanistic underpinnings of FZD7 function in tumor-initiating basal cells remain incompletely understood. FZD7 has been implicated in both canonical WNT/β-catenin signaling and noncanonical pathways that drive cell migration, EMT, and metastasis (4, 5). Given that MMTV-*Wnt1* tumors are exquisitely WNT-dependent and highly sensitive to PORCN inhibitors (45), one plausible model is that FZD7 mediates Wnt1-driven canonical signaling via heterodimerization with LRP6. Our organoid studies, in which the engineered FZD7-LRP6 agonist F7L6 drives β-catenin signaling and organoid growth, are consistent with this view. Yet, we cannot rule out contributions from noncanonical signaling pathways, and the relative importance of these pathways in basal vs. luminal compartments remains an open question. Future studies dissecting downstream signaling events in Fzd7-positive basal cells will be essential for clarifying how FZD7 sustains tumor growth and for identifying additional therapeutic vulnerabilities.

Taken together, these findings establish FZD7 as a marker of tumor-initiating basal cells in TNBC, demonstrate the efficacy of a highly selective FZD7-ADC, and highlight the translational utility of organoid models in preclinical cancer research. By overcoming the limitations of prior WNT/FZD-targeted therapies, this work positions FZD7 as a promising therapeutic entry point in aggressive breast cancers. At the same time, it raises important questions about the precise signaling mechanisms downstream of FZD7 and how they intersect with tumor heterogeneity, lineage hierarchies, and therapy resistance. Addressing these questions will not only refine the rationale for FZD7-targeted therapies but also deepen our understanding of how WNT/FZD biology can be harnessed for clinical benefit.

## Materials and Methods

Detailed materials and methods are provided in *SI Appendix*.

### Generation of MMTV-*Wnt1*;Fzd7^HF7/HF7^ Mouse Line.

MMTV-*Wnt1* mice were obtained from The Jackson Laboratory [B6SJL-Tg(Wnt1)Hev/J, strain #: 002870, RRID:IMSR_JAX:00287] and backcrossed to the HF7 (Fzd7^HF7/HF7^) strain (10) to generate female mice used in these studies. WT (not HF7) MMTV-*Wnt1* mice served as controls where indicated. All animal procedures were done in accordance with protocols approved by the IACUC (protocol number S05387, PI: K. Willert) at the University of California, San Diego.

### Antibodies, Recombinant Proteins, and Small Molecules.

The FZD7 antibody (F7-Ab, working stock concentration 0.1 mg/mL), Wnt mimetic (F7L6), and F7-Ab-MMAE drug conjugate (F7-ADC) were previously described (10, 34). Antibodies used in FC, IHC, immunofluourescence, and immunoblotting and their sources are provided in *SI Appendix*. Wnt3a was purified as previously described (46, 47).

### IHC, IF, and Immunoblotting.

Fixed tumor samples were prepared for IHC and sectioned by the University of California San Diego (UCSD) Tissue Technology Shared Resource (TTSR) as previously described (10). Representative images were taken at 20× magnification using Andor Dragonfly spinning disc confocal microscope system (Oxford Instruments). Immunoblot analyses were performed as previously described (34).

### FC and Cell Sorting.

Tumors were dissociated into single cells as described in *SI Appendix*, labeled with the indicated antibodies, and analyzed or cell-sorted using instrumentation available in the UCSD Human Embryonic Stem Cell Core Facility. Data were analyzed using FlowJo (BD Biosciences).

### Orthotopic Transplantation Immunocompetent Animal Studies.

Five- to six-week-old female C57BL/6 or HF7 mice (The Jackson Laboratory) were used in this study and followed the care and use of laboratory animal guidelines of the NIH. The orthotopic model was established following a previously described protocol (48) with minor modifications as described in *SI Appendix*. Viable tumor cells were isolated from MMTV-*Wnt1*;HF7 mice either unsorted or sorted for hFzd7-high and -low populations using F7-Ab. Under isoflurane-induced anesthesia, mice were injected with 50 µL cell suspensions into the 4th mammary fat pad. Tumor growth was monitored two to three times per week with caliper measurements upon palpation. To test the effect of F7-ADC, as soon as palpable and measurable tumors developed, transplanted mice were randomized into groups based on tumor size and body weight. Treatments of 5 mg/kg F7-ADC or PBS control were delivered twice per week by retro-orbital injection. Human IgG in mouse serum samples was detected using the IgG (Total) Human Uncoated ELISA Kit (Invitrogen #88-50550), as previously described (10). No signals were detected from sera of mice treated with PBS control.

### Establishment of 3D Breast Tumor Organoids, Cell Lines, and Culture Conditions.

Dissociated tumor cells were resuspended at a seeding density of 3,000 to 5,000 cells per 30 µL ECM (Growth factor reduced Matrigel® [Corning]). Media formulations were as described elsewhere (37, 38) and are detailed in *SI Appendix*. Organoids were passaged every 5 to 10 d and kinetic data were captured using an incubated microscope, IncuCyte^®^ S3, which was purchased with funding from a NIH SIG grant (1S10OD02506001). To establish mouse mammary tumor cell lines (C36, C59, C148, etc.), dissociated tumor cells were cultured and allowed to grow for at least 7 to 14 d without replacing medium. Once cells reached near confluence, they were passaged to a fresh plate at split ratios of 1:2 to 1:5. Generally, each line required four passages to become established as a robustly growing cell line. TNBC cells MDA-MB-231 (ATCC; RRID:CVCL 0062) and ovarian cancer cells MA-148 (kindly provided by Prof. Sundaram Ramakrishnan, University of Miami, Miami, FL; RRID:CVCL_AK47) were cultured in RPMI1640 supplemented with 10% FBS and 1% penicillin/streptomycin. MDA-MB-231 FZD7 knockout cell line (clone KO_2G9) was generated using the same procedure as previously described for MA-148 cells (10).

### Confocal Microscopy.

Organoids were collected after ECM depolymerization, fixed and permeabilized, and then incubated with primary antibodies, fluorochrome-conjugated secondary antibodies and DAPI. Images were captured on Andor Dragonfly spinning disc confocal system at 20× magnification.

### Statistical Analyses.

Data were analyzed and graphs were prepared using Prism (GraphPad, San Diego, CA). Student’s *t* test or one-way ANOVA statistical analyses, using All Pairwise Multiple Comparison Procedures (Holm–Sidak method), were performed when comparing two groups in an experiment. Statistical significance was set for a value of *P* ≤ 0.05. Error bars indicate the SEM.

## Supplementary Material

Appendix 01 (PDF)

## Data Availability

All study data are included in the article and/or *SI Appendix*. Previously published data were used for this work (10, 23, 34).
