# Supplementary material for: FZD7 expression marks mammary tumor–initiating cells
Source: Proc Natl Acad Sci U S A. 2025 Nov 11;122(46):e2522465122. doi: 10.1073/pnas.2522465122 (PMC12646316; doi:10.1073/pnas.2522465122)
Supplement: Supplementary file 1 — Appendix 01 (PDF) [file pnas.2522465122.sapp.pdf]

PNAS

Supporting Information for
FZD7 expression marks mammary tumor initiating cells.

Christina C. N. Wu, Naycari De Luna, Erin Hairston, Erin D. Jeffs, Ashley Key, Stephen R. Adams, Sunil J. Advani, Terry Gaasterland, Dennis A. Carson, Karl Willert

Corresponding authors: Dennis A. Carson and Karl Willert
Email: dcarson@health.ucsd.edu, kwillert@health.ucsd.edu

This PDF file includes:

Supporting Text
Figures S1 to S5 with Legends
SI References

Supporting Information Text

SUPPLEMENTAL MATERIALS AND METHODS

Generation of MMTV-Wnt1;Fzd7$^{HF7/HF7}$ mouse line

MMTV-*Wnt1* mice were obtained from The Jackson Laboratory [B6SJL-Tg(*Wnt1*)Hev/J, strain #: 002870, RRID:IMSR_JAX:00287] and backcrossed to the HF7 (*Fzd7*$^{HF7/HF7}$) strain (1). Hemizygous MMTV-*Wnt1* males were bred to HF7 female and pups were genotyped to select for hemizygous MMTV-*Wnt1*;*Fzd7*$^{HF7/HF7}$ mice (Suppl. Fig. 1). Ear punchouts from 3-week-old mice were collected into sterile 0.2 mL PCR tubes containing 50 µL of tissue digestion buffer (50 mM NaOH, 0.2 mM EDTA), heated at 95°C for 90 minutes and neutralized with the addition of 45 µL 0.25M Tris-Cl, pH 7.5, and 5 µL 1M Tris-Cl, pH 7.5. 2 µL of these genomic DNA samples were used for PCR with Fzd7 and Wnt1 primers at 0.5 µM and GoTaq (Promega, M7123). *Fzd7* PCR products were digested with BglII restriction enzyme (NEB, R0144M) at 37°C overnight to detect the modified version of the *Fzd7* gene. *Wnt1* and digested *Fzd7* PCR products were analyzed in a 2.5% agarose gel and imaged with Alpha Imager EC (Alpha Innotech).

Primer sequences

- Fzd7 forward: 5'-GGCGAGATCTGCGTGGGG-3'
- Fzd7 reverse: 5'-CACCATGAAGTAGCAACCCGACA-3'

Expected band size: WT 423 bp, HOMO 329 bp, HET 423 & 329 bp

- Wnt1 forward: 5'-GGACTTGCTTCTCTTCTCATAGCC-3'
- Wnt1 reverse: 5'-CCACACAGGCATAGAGTGTCTGC-3'
- Wnt1 internal control forward: 5'-CAAATGTTGCTTGTCTGGTG-3'
- Wnt1 internal control reverse: 5'-GTCAGTCGAGTGCACAGTTT-3'

Expected band size: tgWnt1 440 bp and internal control 200 bp

Nulliparous females were monitored weekly for the appearance of a palpable nodule in the mammary fat pads. Ages were recorded at the time of tumor identification and tumors were measured twice weekly using a caliper. Tumor volume was determined using the modified ellipsoid formula $(L^2xW)/2$. All animal procedures were done in accordance with protocols approved by the IACUC (protocol number S05387, PI: K. Willert) at the University of California, San Diego.

Antibodies, recombinant proteins and small molecules

The FZD7 antibody (F7-Ab, working stock concentration 0.1 mg/mL), Wnt mimetic (F7L6) and F7-Ab-MMAE drug conjugate (F7-ADC) were previously described (1, 2). Antibodies used in flow cytometry, immunohistochemistry, immunofluourescence and immunoblotting are listed in the table below. Wnt3a was purified as previously described (3, 4). Rspo1-myc was purified from conditioned medium (CM) harvested from HEK293_Rspo1-myc cells (kindly provided by Prof. Xi He, Harvard Medical School) as follows: using an ÄKTA pure 25 L at 4°C, 1 L of CM was passed over a 1 mL HiTrap™ Heparin HP (Cytiva Life Sciences) column, washed with 10 column volumes (CV) with PBS, and then eluted with PBS + 1 M NaCl in a series of steps (10 CVs at 40% elution buffer, 10 CVs at 85%, 10 CVs at 100%). The majority of Rspo1-myc eluted in the 85% step and was detected by immunoblotting at ~40 kDa with mouse anti-Myc (9E10) and by Coomassie staining. Peak fractions were subsequently fractionated on a gel filtration column (Superdex 200 16/60 pg, Cytiva) in PBS. Protein concentrations were estimated by Coomassie staining and adjusted to 100 µg/mL. The following small molecules were purchased from Selleckchem (Houston, TX): Porcupine inhibitor C59 (cat# S7037) and MMAE (cat# S7721).

DESIGNATION	SOURCE	IDENTIFIER	NOTE
Anti-ß-actin mouse mAb	Sigma	A2228	WB 1:5,000
E-Cadherin (CD324) rat mAb (DECMA-1)	Invitrogen/ eBioscience	14-3249-82	IF 1:400

CD24-PE rat anti-mouse mAb, clone M1/69	eBioscience	12-0242-82	FC 1:100
CD45 PE/Cy7 rat anti-mouse mAb clone 30-F11	eBioscience	25-0451-82	FC 1:100
CD49f (Integrin alpha 6) APC-eFluor 780 rat anti-mouse/human mAb, clone eBioGoH3 (GoH3)	Invitrogen/ eBioscience	47-0495-82	FC 1:100
EpCAM (CD326) rat anti-mouse mAb, clone G8.8	Invitrogen, Biolegend,	11-5791-82 (FITC)<br>18231 (BV510)	FC 1:100<br>IF 1:400
F7-Ab	Willert lab	h1791	FC 1:100 (AF647 conjugatec<br>IF 1:400~1,000<br>WB 1:2,000
Goat anti-Human IgG (H+L) Cross-Adsorbed Secondary Antibody, Fluor™ 647	Fisher Scientific	A-21445	IF: 1:1,000
Goat anti-Rat IgG (H+L) Cross-Adsorbed Secondary Antibody, Fluor™ 647	Invitrogen	A-21247	IF 1:500~1:2,000
Goat anti-Rabbit IgG (H+L) Cross-Adsorbed Secondary Antibody, Alexa Fluor™ 488	Invitrogen	A-11008	IF 1:500~2,000
Goat anti-Rabbit IgG (H+L) Cross-Adsorbed Secondary Antibody, Alexa Fluor™ 647	Invitrogen	A-21245	IF 1:500~2,000
Goat anti-human IgG-HRP	Southern Biotech	2040-05	WB 1:20,000
Anti-mouse IgG-HRP	Southern Biotech	1031-05	WB 1:50,000

Immunohistochemistry (IHC) and Immunofluorescence (IF)

Tumor samples were fixed in 4% PFA, washed with PBS, and stored in either 70% EtOH for paraffin section or 30% sucrose clearing solution for 2 days at 4°C for OCT section. H&E staining and IHC of paraffin sections were performed by UCSD Tissue Technology Shared Resource (TTSR) as previously described (Do et al., 2022). Images of tumor IHC (Fig. S2A) were captured using EVOS M5000 imaging system (Invitrogen). Cleared tumor samples were embedded in OCT at -80°C and sectioned slides were stored at -20°C until ready for IF staining. Frozen slides were thawed at room temperature (RT) for 30 min to 2 hr, and OCT was removed from the edges of tumor by Q-tips dipped in PBS. Samples were circled with a PAP pen to create a hydrophobic barrier and allowed to dry. Slides were washed 3 times each with PBS for 2-5 min, permeabilized in IF blocking solution containing 0.1% Triton, 1% BSA and 20% goat serum in PBS at RT for 1hr, incubated with primary antibodies at 4°C overnight, washed 3x, incubated with secondary antibodies and DAPI (Cell Signaling Technology, 4083S, 5 mg/mL diluted 1:400) at RT for 2hr or 4°C overnight, washed 3x, mounted with Prolong Gold Antifade Mountant (Invitrogen, P36930) and a coverslip was placed and allowed to dry overnight at RT in the dark at 4°C. Representative images were taken at 20X magnification using Andor Dragonfly spinning disc confocal microscope system (Oxford Instruments).

Preparation of tumor-cell suspension for flow cytometry and cell sorting

Tumors were finely minced with sterile autoclaved razor blades to 1–3 mm^3 sized pieces and digested in DMEM/F12 (Gibco #11330-032) containing 5% FBS, 1x collagenase/hyaluronidase mixture (5) for 1 h at 37°C with vortexing every 15 min and shaking at 180–200 rpm on a platform shaker. Digested tumor slushes were neutralized with the addition of 2-fold volume of complete medium, centrifuged, and red blood cells removed with ACK lysis buffer (Gibco #A10492-01). Single cells were obtained from further incubation of the pellet resuspended in “TeDD” dissociation solution (TrypLE Express [ThermoFisher, Waltham, MA], 10 U/mL DNase I [ThermoScientific #EN0521] and 5 mg/mL Dispase [Sigma #4942078001]) for 3–5 min at 37°C, washed, resuspended in ice-cold staining media (StM: 2% FBS and 3 mM EDTA in HBSS-/- [Gibco #14175-079] or PBS), and filtered through 35–40 µm cell strainer (BD Biosciences, Franklin Lakes, NJ, USA). To preserve hFzd7 epitope on the cell surface, mild dissociation reagents, here TrypLE Express, and short incubation times were used. Final cell counts were determined using 1:1 dilution in Trypan Blue Solution, 0.4% (Thermo Fisher Scientific) and counted on a hemocytometer. Cells were blocked with human Fc and mouse Fc for 30 min on ice and stained with the following fluorochrome-conjugated antibodies: EpCAM-FITC or -BV510, CD24-PE, F7-Ab-AF647, CD45-PE-Cy7, and Cd49f-eFluor AF780. After 30–45 min incubation on ice in the dark, cells were washed with ice-cold StM, centrifuged, and final suspension in StM with DAPI (1:6,000 dilution, i.e., 0.83 µg/mL). Cells were sorted on a FACS Aria II (BD) or immune-profiled on a LSRFortessa X20 (BD Biosciences), and data were analyzed using FlowJo (BD Biosciences). Dissociated tumor cells were gated for non-debris singlets and live immune cells (marked by CD45) were excluded. Fluorescence minus one (FMO) controls were used to establish appropriate gating, especially for sorting of hFzd7 expressing cells with AF647-FMO compared to full-stained samples.

Orthotopic transplantation immunocompetent animal studies

Five- to 6-wk-old female C57BL/6 mice (The Jackson Laboratory) were used in this study unless otherwise indicated and followed the care and use of laboratory animal guidelines of the National Institutes of Health (NIH). Mice were housed in laminar-flow cabinets under specific pathogen-free conditions and fed ad libitum. The orthotopic model was established following a previously described protocol (6) with minor modifications. Briefly, viable tumor cells were isolated from MMTV-Wnt1;HF7 mice either unsorted or sorted for hFzd7-high and -low populations using F7-Ab. FACS-purified or cultured cell lines were resuspended in PBS and mixed with high concentration Matrigel® Matrix (Corning, 354262) at a 1:1 ratio, aliquoted to tubes for individual mouse injections and kept on ice. Under isoflurane-induced anesthesia, mice were injected with 50 µL cells into the 4th mammary fat pad (MFP) using a 27G needle. Tumor growth was monitored 2–3 times per week with caliper measurements upon palpation as described above. To test the effect of F7-ADC, as soon as palpable and measurable tumors developed, transplanted mice were randomized into groups based on tumor size and body weight. Treatments of 5 mg/kg F7-ADC or PBS control were delivered twice per week by retro-orbital injection. Small amounts of blood were collected via submandibular vein approximately 24hr after the 2nd and 4th treatments. Collected sera were centrifuged at the maximum speed for 10 min at -20°C. After 7 doses, mice were euthanized and tissues collected. Tumors were processed into single cells for flow cytometry, and sera were evaluated for F7-ADC levels.

Human IgG ELISA

Detection of human IgG in mouse serum samples was performed using the IgG (Total) Human Uncoated ELISA Kit (Invitrogen #88-50550) as previously described (1) with minor modifications: detection antibody (50 µL/well) was coated in high binding, half area, 96-well plate (Corning Costar #3690), hIgG standards started from 100 µg/mL with 2-fold serial dilutions, sera samples diluted at 1:1,000, and stop solution used 1M H_3PO_4. $OD_{450\text{-}570}$ values were determined using the Infinite® 200 Pro microplate reader (Tecan). No signals were detected from sera of mice treated with PBS control.

Establishment of 3D breast tumor organoids, cell lines and culture conditions

Tumor cells were resuspended in extracellular matrix (ECM) composed of PBS and growth factor reduced Matrigel (Corning, 356231) or Cultrex Type II BME (R&D Systems, 3533-005) at 1:1 ratio at a seeding density of 3,000–5,000 cells per 30 µL ECM. A small animal heating pad (K&H Pet Products, 25 Watts, 9x12 inches) was utilized when plating aliquots of the cell suspension on pre-heated cell culture plates to improve doming efficiency. After seeding 20–24 domes, plates were inverted and placed in a cell culture incubator (5% CO_2, 37°C) for 10–15 minutes to prevent cell adhesion on the bottom of the plate and to facilitate ECM solidification. Once the ECM domes solidified, plates were flipped upright again and an appropriate volume of mammary gland organoid medium (MG-OM, see table below) was added to each well, i.e., 750 µL in 24-well plates and 3 mL in 6-well plates. MG-OM formulation was modified from (7, 8) without fetal bovine serum. 3D cultures were grown in MG-OM containing 10 µM Rho-kinase inhibitor Y27632 (ROCKi) only for the first 7 days and OM was replaced every 3–7 days. Organoids were passaged every 5–10 days. ECM domes were rinsed with PBS twice, collected with ice-cold Cell Recovery Solution (Corning. CLS354253; 1 mL per 9–12 domes) into conical tubes and incubated on ice for 15–30 min to depolymerize the ECM. After centrifugation, organoid pellets were dissociated in 1 mL pre-warmed TrypLE Express, incubated at 37°C for 30 sec to 1 minute, neutralized with equal volume of bOM, pipetted 15 times and filtered through 35 µm FACS tube strainer caps. Pellets were washed with PBS once and resuspended in ice-cold PBS, counted, resuspended in PBS:ECM (1:1 ratio) and seeded at 3,000 cells per dome, as mentioned above.

Organoid Medium (OM) Component:	Company	Catalog No.	Final Conc.	MG-OM	OM/S
Advanced DMEM/F12	Gibco	12634028	1X	bOM	bOM
GlutaMax	Gibco	35050-061	1X		
HEPES	Gibco	15630106 or 15630080/SW	10 mM		
Penicillin/Streptomycin	Gibco	15140-122	100 U/mL		
B-27 supplement	Gibco	17504044	50X	1X	1X
R-spondin 1 (RSPO)	In-house production		~100 ng/mL	0.25%	0.25%
rhEGF	Peprotech	AF-100-15	10 µg/mL	5 ng/mL	10 ng/mL
FGF-7 (KGF)	Peprotech	100-19	10 µg/mL	5 ng/mL	—
FGF-10	Peprotech	100-26	50 µg/mL	10 ng/mL	—
Neuregulin 1 (NRG-1, Heregulin-ß1)	Peprotech	100-03	500 µg/mL	100 ng/mL	—
Y-27632 (Rockout, RhoK inhibitor), first 7 days	BioVision	2342-5	10 mM	10 µM	10 µM
FBS			100%	—	2%

To establish mouse mammary tumor cell lines (C36, C59, C148, etc.), dissociated tumor cells were cultured in DMEM/F-12 supplemented with 10% FBS and 1% penicillin/streptomycin at a density of $2x10^6$ cells per 10 cm^2 plate or $3x10^5$ cells per well in a 6-well plate and allowed to grow for at least 7–14 days without replacing medium. Once cells reached near confluence, they were trypsinized and passaged to a fresh plate at split ratios of 1:2 to 1:5. Generally, each line required 4 passages to become established as a robustly growing cell line.

Triple negative breast cancer (TNBC) cells MDA-MB-231 (ATCC; RRID:CVCL 0062) and ovarian cancer cells MA-148 (kindly provided by Prof. Sundaram Ramakrishnan, University of Miami, Miami, FL; RRID:CVCL_AK47) were cultured in RPMI1640 supplemented with 10% FBS and 1% penicillin/streptomycin. MDA-MB-231 *FZD7* knockout cell line (clone KO_2G9) was generated using the same procedure as previously described for MA-148 cells (1). For the F7-ADC

treatments, MDA-MB-231 cells were seeded at 3,000 cells/well, while MDA-MB-231 FZD7KO cells were seeded at 6,000 cells/well to accommodate for the slower growth rate seen in MDA-MB-231 KO cells, and incubated overnight in a humidified 37°C/CO_2 incubator. Cells were treated with F7-ADC at the indicated concentrations, and 3 days later, fresh media with ADC were added. 4 days later, cell viability was assayed using CellTiter-Glo 2.0 reagent according to manufacturer's instructions (Promega®).

Tumor organoids were seeded at 3,000 cells per 30 µL dome, one dome per well in 24-well plates, and treated each well with 750 µL of OM/S containing 2% FBS and various concentrations of C59 and F7L6. The vehicle control was 0.1% (*v*/*v*) DMSO and PBS in culture medium. To evaluate hFzd7 Ab activity on organoid growth, F7-Ab and F7-ADC were diluted in MG-OM at a final concentration of 100 nM and added to organoid cultures 3 days after seeding. In addition, MMAE at 1 µM was used as a positive control for toxicity. Organoid cultures were maintained for up to 7 days without media replacement. To quantify organoid formation and their growth kinetics, IncuCyte® S3 Live-Cell Analysis System (Sartorius AG) was employed. Organoids were imaged using InCuCyte® bright field (BF) at 4X magnification with z-stack at ~ 2 mm at an interval ranging from 8hr to 24hr and measured by outlining organoid clusters with the Organoid Analysis Module. This publication includes data generated at the UCSD Human Embryonic Stem Cell Core Facility using the IncuCyte® S3, which was purchased with funding from a NIH SIG grant (1S10OD02506001).

Confocal microscopy

Organoids were collected after ECM depolymerization as described above and centrifuged at RCF of 800 for 5 min to prevent shape disruption. P1000 tips cut to widen the tip opening were coated with cold 3% BSA in PBS to prevent loss of organoids due to sticking to the plastic. Organoids were fixed in 4% PFA at 4°C for 30-45 min, washed with ice-cold PBS, blocked and permeabilized with IF blocking solution at 4°C for at least 30 min, incubated with primary antibodies at 4°C overnight followed by 3 washed with 2% BSA in PBS and fluorochrome-conjugated secondary antibodies and DAPI for 2hr or overnight (9, 10). Samples were mounted in either ProLong™ Gold Antifade Mountant (Invitrogen P36930) or Fructose-Glycerol solution containing 60% (*v/v)* glycerol and 2.5M fructose (9) and stored in the dark at 4°C for up to 1 week or -20°C for up to 6 months. Images were captured on Andor Dragonfly spinning disc confocal system at 20X magnification.

Immunoblot

Immunoblot analyses were performed as previously described (2).

Statistical analyses

Data were analyzed and graphs were prepared using Prism (GraphPad, San Diego, CA). Student's *t*-test or one-way ANOVA statistical analyses, using All Pairwise Multiple Comparison Procedures (Holm-Sidak method), were performed when comparing two groups in an experiment. Statistical significance was set for a value of $p \leq 0.05$. Error bars indicate the SEM.

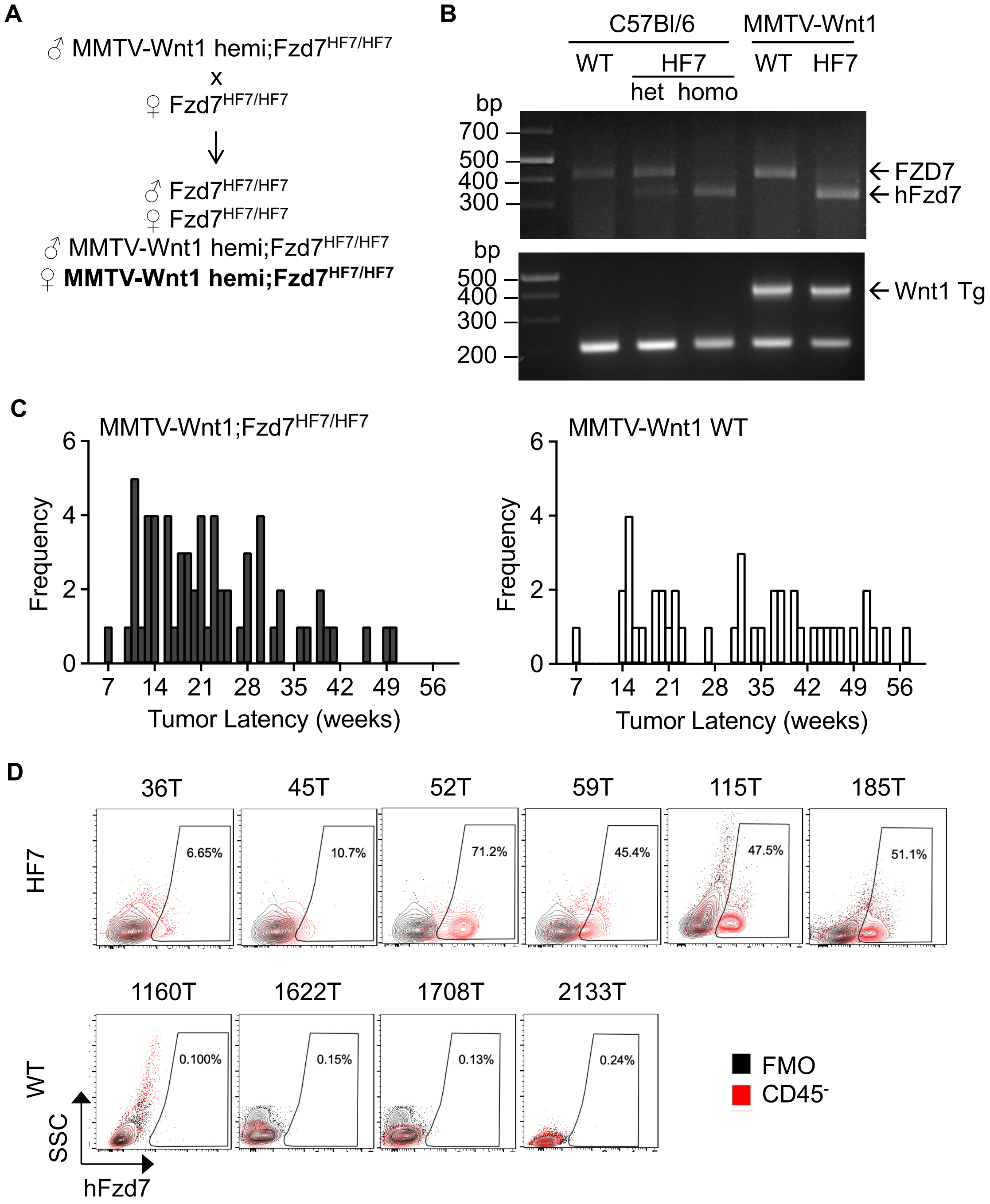

Fig. S1. Description of the mouse mammary tumor model. A. Schematic of mouse crosses. The HF7 allele was introduced into the MMTV-Wnt1 background. The MMTV-Wnt1 transgene is maintained in hemizygous state, because mammary glands of females carrying this transgenes are hyperplastic and fail to produce milk. Females carrying the MMTV-Wnt1 transgene and homozygous for the HF7 allele are used for tumor studies. Tumors from wildtype (not HF7) females carrying the MMTV-Wnt1 transgene served as a negative control for detection of hFzd7 protein using the F7-Ab. B. PCR-based genotyping of HF7 allele (top) and the MMTV-Wnt1 transgene (bottom). C. Tumor latency in MMTV-Wnt1 females. D. Flow cytometry of independent mammary

tumors from MMTV-Wnt1 mice. Fzd7 expression is variable and only detected in tumors derived from HF7 mice.

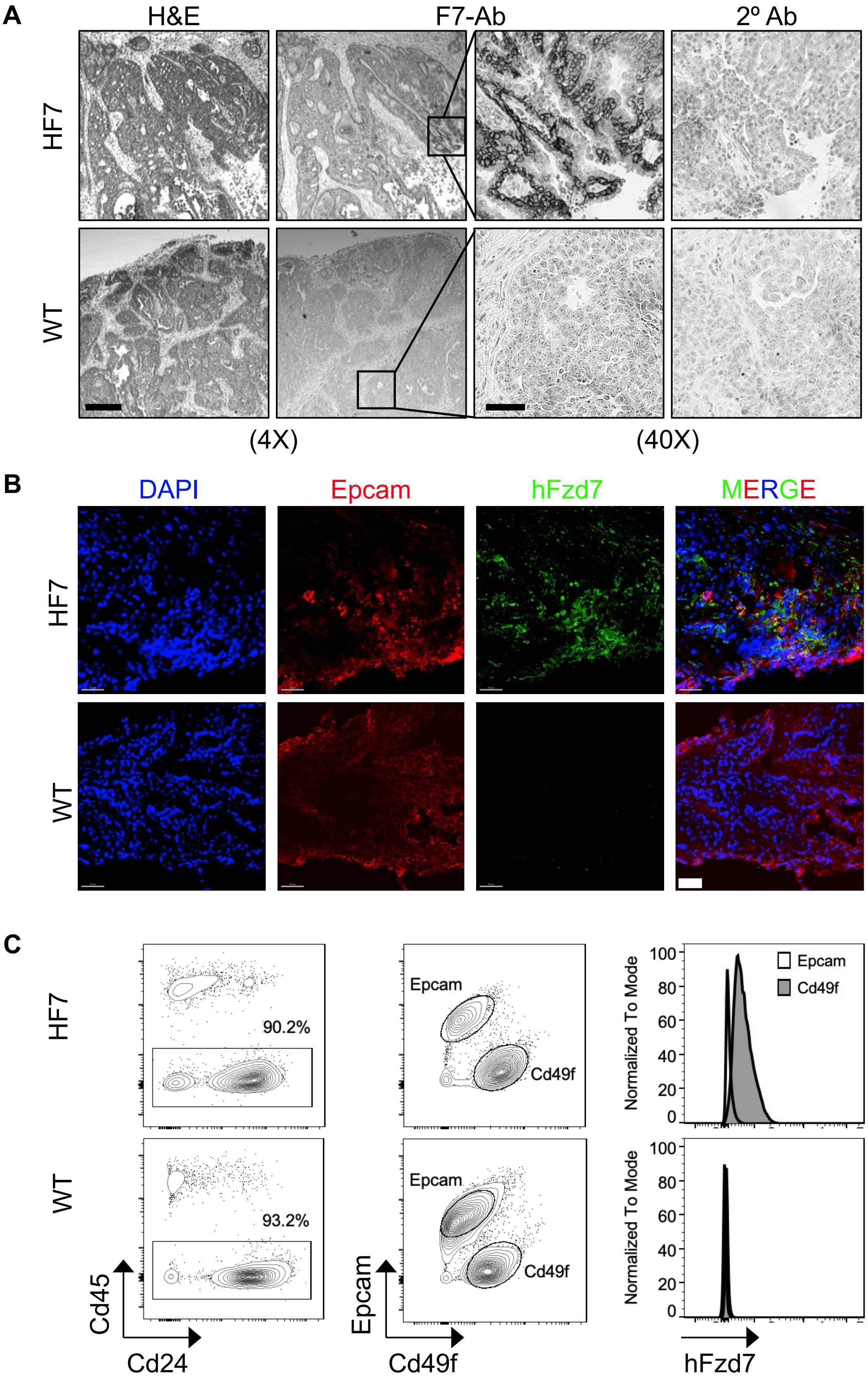
A
H&E
F7-Ab
2º Ab
HF7
WT
(4X)
(40X)
B
DAPI
Epcam
hFzd7
MERGE
HF7
WT
C
HF7
WT
90.2%
93.2%
Cd45
Cd24
Epcam
Cd49f
Epcam
Cd49f
Epcam
Cd49f
Epcam
Cd49f
Normalized To Mode
100
80
60
40
20
0
Epcam
Cd49f
hFzd7

Fig. S2. Fzd7 expression in MMTV-Wnt1 mammary gland tumors. A. Cryo-sections of MMTV-Wnt1 tumors derived from HF7 or wildtype (WT) mice were stained with hematoxylin and eosin (H&E) to visualize tissue structures or with a hFzd7-specific antibody (F7-Ab). Scale bar = 250 μm in 4X and 50 μm in 40X magnification. B. Immunofluorescence microscopy of tumors derived from HF7 or WT mice reveals that Epcam, a luminal marker, and hFzd7 are expressed in distinct cell populations. Scale bar = 30 μm. C. Flow cytometric analysis reveals the presence of luminal and basal compartments labeled by Epcam and Cd49f, respectively. hFzd7 expression is only detected in Cd49f basal cells.

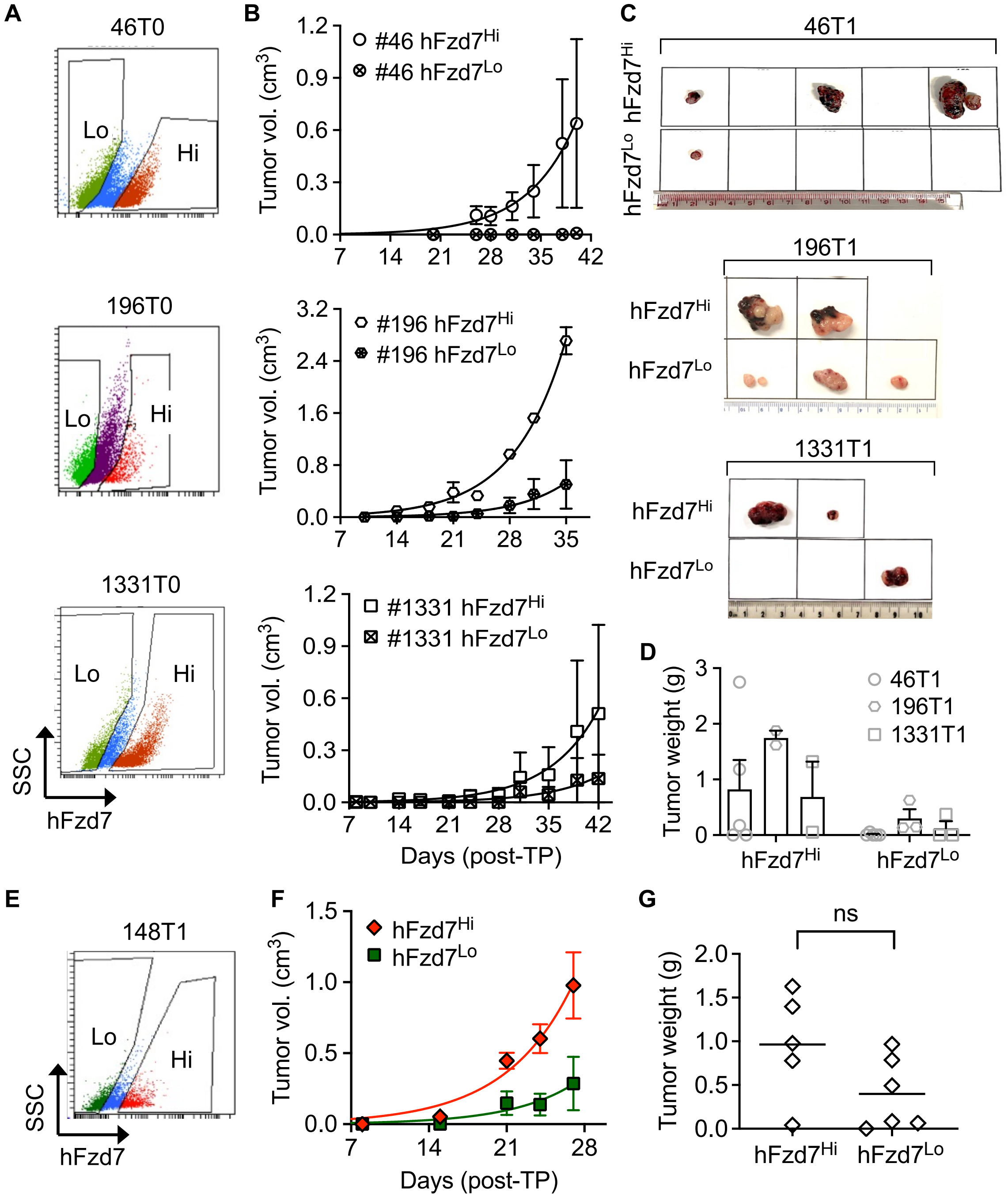

Fig. S3. Fzd7 expressing cells harbor tumorigenic potential (other tumor lines and secondary transplant). A-D. Serial transplantation of primary tumors isolated from 3 independent mice. A. Dissociated tumor cells from 3 independent tumors were depleted for Cd45 and sorted using the fluorescently labelled F7-Ab to isolate hFzd7^{Hi} and hFzd7^{Lo} populations. B. hFzd7^{Hi} and hFzd7^{Lo} populations (10^4 cells/mouse) were orthotopically transplanted into the 4th mammary fat pad of C57BL/6 (n=7, 46T0; n=2-3, 196T0; n=2-3, 1331T0), and tumor volumes were quantified by caliper measurements. C. Images of tumors isolated from mice transplanted with either hFzd7^{Hi} and hFzd7^{Lo} cells as shown in panel B. Bars represent mean $\pm$ SEM. D. Quantitation of tumor weights from mice transplanted with either hFzd7^{Hi} and hFzd7^{Lo} cells as shown in panel C. E-G. Secondary tumor transplantation. E. A transplanted tumor (148T1) was dissociated to single cells, depleted for Cd45 and sorted to isolate hFzd7^{Hi} and hFzd7^{Lo} populations. F. hFzd7^{Hi} and hFzd7^{Lo} populations

from 148T1 (10^4 cells/mouse) were orthotopically transplanted into the 4th mammary fat pad of C57BL/6 (n=5-6), and tumor volumes were quantified by caliper measurements. G. Quantitation of tumor weights from mice transplanted with either $hFzd7^{Hi}$ and $hFzd7^{Lo}$ cells. Error bars indicate SEM. ns, no statistical significance by unpaired two-tailed t test with Welch's correction.

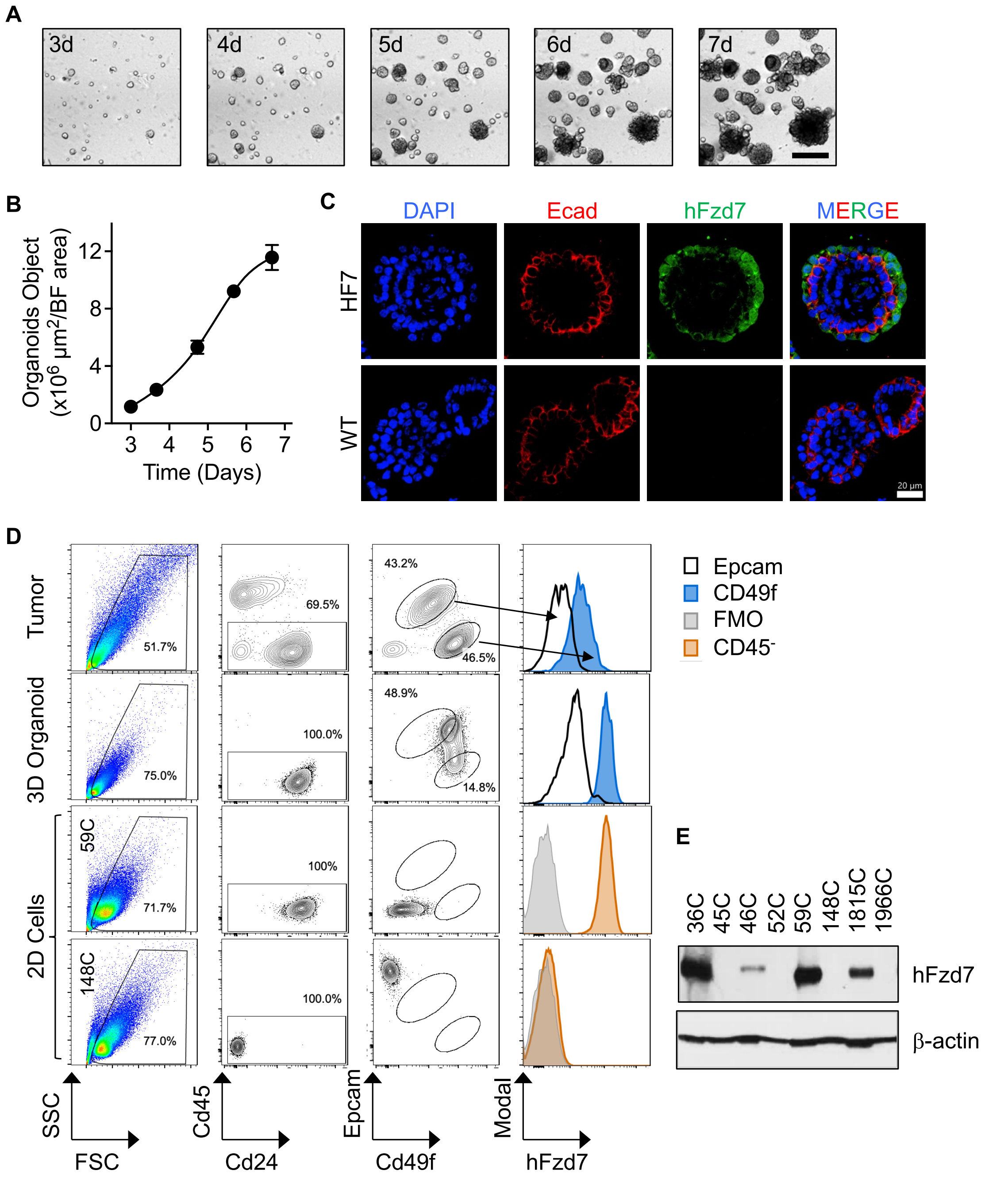

Fig. S4. Establishing mammary tumor organoids. A. Dissociated tumor cells were resuspended in a 3-dimensional extracellular matrix and their growth was tracked over 7 days. Scale bar = 0.3 mm. Representative images are shown from triplicate. Similar results are observed from at least three independent experiments. B. Quantitation of organoid growth using an incubated microscope (Incucyte®). Error bars indicate SEM. C. Immunofluorescence of tumor organoids derived from HF7 and WT MMTV-Wnt1 mice. Scale bar = 20 μm. D. Flow cytometry of dissociated MMTV-Wnt1 tumors, organoids and cell lines. Organoids display a similar heterogeneity as tumors, with co-mingling of basal and luminal populations. In contrast, 2-D cell lines derived from these tumors lack this cellular heterogeneity. E. Immunoblot of multiple cell lines derived from MMTV-Wnt1;HF7 tumors indicates that some cell lines lack hFzd7 expression.

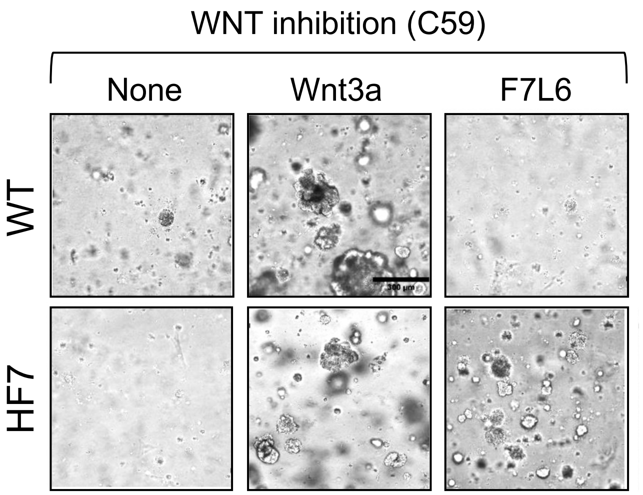

Fig. S5. Tumor organoid growth requires Wnt signaling. Tumor organoids derived from HF7 or WT MMTV-Wnt1 tumors were treated with PORCN inhibitor C59 and with either Wnt3a or F7L6.

SI References

1. M. Do *et al.*, A FZD7-specific Antibody-Drug Conjugate Induces Ovarian Tumor Regression in Preclinical Models. *Mol Cancer Ther* **21**, 113-124 (2022).
2. D. Gumber *et al.*, Selective activation of FZD7 promotes mesendodermal differentiation of human pluripotent stem cells. *Elife* **9** (2020).
3. K. Willert *et al.*, Wnt proteins are lipid-modified and can act as stem cell growth factors. *Nature* **423**, 448-452 (2003).
4. K. H. Willert, Isolation and application of bioactive Wnt proteins. *Methods in molecular biology* **468**, 17-29 (2008).
5. L. Rodriguez de la Fuente, A. M. K. Law, D. Gallego-Ortega, F. Valdes-Mora, Tumor dissociation of highly viable cell suspensions for single-cell omic analyses in mouse models of breast cancer. *STAR Protoc* **2**, 100841 (2021).
6. A. V. Paschall, K. Liu, An Orthotopic Mouse Model of Spontaneous Breast Cancer Metastasis. *J Vis Exp* 10.3791/54040 (2016).
7. J. M. Rosenbluth *et al.*, Organoid cultures from normal and cancer-prone human breast tissues preserve complex epithelial lineages. *Nat Commun* **11**, 1711 (2020).
8. N. Sachs *et al.*, A Living Biobank of Breast Cancer Organoids Captures Disease Heterogeneity. *Cell* **172**, 373-386 e310 (2018).
9. J. F. Dekkers *et al.*, High-resolution 3D imaging of fixed and cleared organoids. *Nat Protoc* **14**, 1756-1771 (2019).
10. K. V. Nguyen-Ngoc *et al.*, 3D culture assays of murine mammary branching morphogenesis and epithelial invasion. *Methods in molecular biology* **1189**, 135-162 (2015).
